# Supplementary material for: Comparing the effectiveness of negative-pressure barrier devices in providing air clearance to prevent aerosol transmission
Source: PLoS One. 2021 Apr 21;16(4):e0250213. doi: 10.1371/journal.pone.0250213 (PMC8059829; doi:10.1371/journal.pone.0250213)
Supplement: Attachment — Submitted filename: Response to reviewers.docx [file pone.0250213.s002.docx]

**Comment from the reviewer：**

Reviewer #1: This is an interesting article regarding the effectiveness of negative pressure barrier devices in providing air clearance to prevent aerosol transmission.
The topic is promising
**Our response:**

We are very pleased that you found our study interesting and informative. We have revised the manuscript thoroughly following your valuable suggestions. We hope the revised manuscript is now satisfactory.

**Reviewer’s comment:**

- The whole manuscript should be edited for English-language usage

**Our response:**

We apologize for the language in the manuscript. The document has now been proofread by an professional English language editor.

**Reviewer’s comment:**

- In the introduction the authors must describe the main aim avoiding too detailed descriptions that should be reported in the methods (primary and secondary etc.)

**Our response:**

Dear reviewer, we appreciate your valuable suggestion and adjusted the paragraph following your comment. We removed the part at the end of the introduction, which was more suitable in the study design and setting to improve the flow of the context.

The paragraph: ”Atomized glycerol was selected as a tracer gas and poly alpha olefins (PAO, diameter, 0.5–0.7 μm) was selected for aerosol concentration detection, based on previously published aerosol studies.^24-26^ The tracer gas with such a diameter was demonstrated to be best correlated with the aerosol droplet nuclei movement in an enclosed space.^27^ Our primary goal was to evaluate the effectiveness of each aerosol clearing device (AB, plastic aerosol bag, wall suction, and HEPA filter evacuator). Our secondary goal was to……” was removed from the introduction.

**Reviewer’s comment:**

- Is there a study period?
**Our response:**

Thank you for your query. This simulation experiment was conducted during 2020. The study period was April 20^th^ to August 26^th^ 2020.

**Reviewer’s comment:**

- In the introduction the authors give too many references to describe a concept (2-10). Should be reduced by adding the following guidelines on the use of laparoscopy in surgery (and the connection with aerosolization)
The risk of COVID-19 transmission by laparoscopic smoke may be lower than for laparotomy: a narrative review. Surg Endosc. 2020 Aug;34(8):3298-3305. doi: 10.1007/s00464-020-07652-y
Italian society of colorectal surgery recommendations for good clinical practice in colorectal surgery during the novel coronavirus pandemic. Tech Coloproctol. 2020 Jun;24(6):501-505. doi: 10.1007/s10151-020-02209-6
A Low-cost, Safe, and Effective Method for Smoke Evacuation in Laparoscopic Surgery for Suspected Coronavirus Patients. Ann Surg. 2020 Jul;272(1):e7-e8. doi: 10.1097/SLA.0000000000003965

**Our response:**

We appreciate your valuable comments and have revised our references for each concept in this regard. Furthermore, three important studies that link the procedures and aerosolization have been added to the Reference list.

**Reviewer’s comment:**

- Were any patients involved? How many?

**Our response:**

Since our practice acquired a negative-pressure barrier device on January 19^th^ 2021, a total of 32 patients have received tracheal intubation using the negative-pressure device.

**Reviewer’s comment:**

- The authors must clarify the methodology of the study

**Our response:**

Dear reviewer, we appreciate your important suggestion and had clarify the experiment setting and methodology of the study in the **study design and settings** to make sure the readers can understand the flow field environment for aerosol visualization and the process for aerosol concentration detection.
