# Supplementary material for: Comparing the effectiveness of negative-pressure barrier devices in providing air clearance to prevent aerosol transmission
Source: PLoS One. 2021 Apr 21;16(4):e0250213. doi: 10.1371/journal.pone.0250213 (PMC8059829; doi:10.1371/journal.pone.0250213)
Supplement: Attachment — Submitted filename: Response to reviewers.docx [file pone.0250213.s003.docx]

**Comment from the reviewer：**

**Reviewer’s comment:**

- In the introduction the authors give too many references to describe a concept (2-10). Should be reduced by adding the following guidelines on the use of laparoscopy in surgery (and the connection with aerosolization)
The risk of COVID-19 transmission by laparoscopic smoke may be lower than for laparotomy: a narrative review. Surg Endosc. 2020 Aug;34(8):3298-3305. doi: 10.1007/s00464-020-07652-y
A Low-cost, Safe, and Effective Method for Smoke Evacuation in Laparoscopic Surgery for Suspected Coronavirus Patients. Ann Surg. 2020 Jul;272(1):e7-e8. doi: 10.1097/SLA.0000000000003965

**Our response:**

We appreciate your valuable comments and have revised our references for each concept in this regard. Furthermore, these important studies that link the procedures and aerosolization have been added to the Reference list (10 and 20).
